# Recent Developments in Ventricular Assist Device Therapy

**DOI:** 10.31083/RCM25440

**Published:** 2025-01-14

**Authors:** Angel Moctezuma-Ramirez, Haseeb Mohammed, Austin Hughes, Abdelmotagaly Elgalad

**Affiliations:** ^1^Center for Preclinical Surgical & Interventional Research, The Texas Heart Institute, Houston, TX 77030, USA; ^2^Texas A&M School of Medicine, Bryan, TX 77843, USA; ^3^The University of Texas Health Science Center at Houston, Houston, TX 77054, USA

**Keywords:** LVAD, minimally invasive surgery, pediatric VAD, RVAD support, wireless LVAD, cost-effective VAD

## Abstract

The evolution of left ventricular assist devices (LVADs) from large, pulsatile systems to compact, continuous-flow pumps has significantly improved implantation outcomes and patient mobility. Minimally invasive surgical techniques have emerged that offer reduced morbidity and enhanced recovery for LVAD recipients. Innovations in wireless power transfer technologies aim to mitigate driveline-related complications, enhancing patient safety and quality of life. Pediatric ventricular assist devices (VADs) remain a critical unmet need; challenges in developing pediatric VADs include device sizing and managing congenital heart disease. Advances in LVAD technology adapted for use in right ventricular assist devices (RVADs) make possible the effective management of right ventricular failure in patients with acute cardiac conditions or congenital heart defects. To address disparities in mechanical circulatory support (MCS) access, cost-effective VAD designs have been developed internationally. The Vitalmex device from Mexico City combines pulsatile-flow technology with a paracorporeal design, utilizing cost-effective materials like silicone-elastic and titanium, and features a reusable pump housing to minimize manufacturing and operational costs. Romanian researchers have used advanced mathematical modeling and three-dimensional (3D) printing to produce a rim-driven, hubless axial-flow pump, achieving efficient blood flow with a compact design that includes a wireless power supply to reduce infection risk. In conclusion, MCS continues to advance with technological innovation and global collaboration. Ongoing efforts are essential to optimize outcomes, expand indications, and improve access to life-saving therapies worldwide.

## 1. Introduction

Heart failure (HF) represents a growing public health issue, affecting about 6.7 million Americans today and projected to affect up to 8.5 million by 2030 [1]. Although HF predominantly affects older adults, its prevalence is increasing across all age groups. The lifetime risk of developing HF has risen to approximately 24%, indicating that about one in four individuals will have some degree of HF during their lifetime. For those with severe end-stage HF, heart transplantation remains the ideal therapeutic option, but it is suitable only for a small subset of patients due to factors such as organ availability and eligibility criteria.

For HF patients, heart transplantation was historically the only viable treatment option. However, the scarcity of donor hearts and the complexities associated with transplantation have spurred the development of alternative therapies, including mechanical circulatory support (MCS) [2]. MCS with left ventricular assist devices (LVADs) has emerged as a crucial therapy for advanced HF, offering a lifeline to patients who are not candidates for transplantation or are awaiting donor hearts [3].

LVADs, designed to support the failing heart’s function, serve multiple roles: bridging patients to transplantation, providing destination therapy for those ineligible for transplantation, and even facilitating recovery in a few cases. The adoption of LVADs has surged, with continuous-flow devices predominating because they are smaller and have greater durability than earlier, pulsatile models [4]. This shift from pulsatile to continuous-flow devices has significantly improved patient outcomes, with third-generation devices like the HeartMate 3 demonstrating superior performance and fewer complications than their predecessors, as evidenced by trials like MOMENTUM 3 [5].

However, these devices are not without complications, some of which can profoundly affect patient outcomes. Among the most serious complications is thromboembolism—both pump thrombosis and systemic embolization [6]. Pump thrombosis involves clot formation within the device, potentially leading to malfunction and compromised blood flow, while systemic embolization involves clots dislodging and traveling through the bloodstream, potentially causing stroke and other ischemic events. As a result, patients with LVADs are also at elevated risk for neurological events, including strokes, necessitating optimized anticoagulation management and vigilant monitoring [7].

To prevent thrombotic complications, LVAD-supported patients undergo continuous anticoagulation therapy. However, anticoagulation increases the risk of bleeding, with gastrointestinal bleeding being particularly common and severe. This type of bleeding is often related to a combination of factors that can interfere with hemocompatibility, such as arteriovenous malformations, and the risk of bleeding is further compounded by anticoagulation. Gastrointestinal bleeding frequently necessitates interventions such as blood transfusion and surgery. Therefore, careful management of anticoagulation therapy is crucial to balance the risks of bleeding and thrombosis in LVAD-supported patients [8].

Infection is also a major concern, especially at the exit site of the driveline that connects many LVADs to an external power source, as the driveline can serve as a bacterial entry point. Such infections are potentially life-threatening. Right HF is another significant risk because LVADs increase blood flow from the left ventricle, placing pressure on the right ventricle and potentially necessitating additional support and careful hemodynamic monitoring [9]. Mechanical failures of the LVAD, such as pump stoppage and electrical issues, pose risks that necessitate regular maintenance and prompt troubleshooting. Hemolysis, the destruction of red blood cells by the LVAD’s mechanical forces, can lead to anemia and jaundice; preventing hemolysis requires close monitoring and possible adjustments in device settings or anticoagulation therapy.

**Fig. 1.**
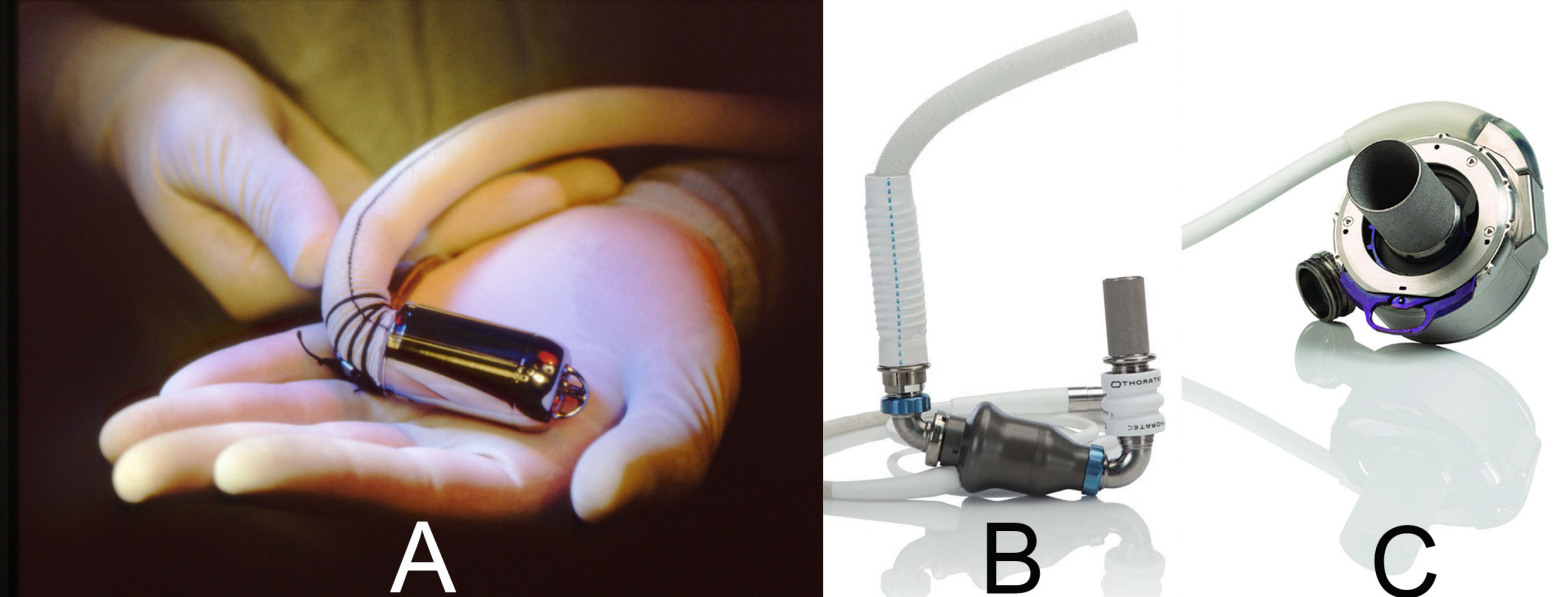
**Notable left ventricular assist devices currently in use**. (A) Jarvik 2000. (B) HeartMate II. (C) HeartMate 3.

Recent advancements in LVAD therapy have prominently featured the HeartMate 3, HeartMate II, and Jarvik 2000 (although its use has been in decline) (Fig. 1). The HeartMate 3, introduced in 2015, has become the most widely used LVAD due to its superior patient outcomes and durability. The MOMENTUM 3 trial reported a 2-year survival rate of 82% and a 2-year freedom from stroke rate of 69% with the HeartMate 3 [10]. This device is available in the United States, Canada, Germany, France, the United Kingdom, Italy, Spain, Australia, Japan, and South Korea. The HeartMate II, with its continuous-flow design, has been associated with a 2-year survival rate of 74% but also with a higher incidence of pump thrombosis than the HeartMate 3 [11]. The HeartMate II is available in the same countries as the HeartMate 3. The Jarvik 2000, a pulsatile-flow LVAD, is used in specific scenarios where pulsatility is beneficial. A multicenter, prospective study in the United States showed a 1-year survival rate of approximately 60% with the Jarvik 2000 [12]. It is available in the United States, Canada, Japan, Germany, the United Kingdom, Italy, and Spain.

The ongoing evolution of LVAD technology, coupled with research aimed at further enhancing outcomes and minimizing complications, holds considerable promise for the future of HF management. Here, we explore improvements in design and materials for LVADs, technology to make them wireless, minimally invasive techniques for their implantation, right ventricular assist devices (RVADs), and innovations to make more affordable LVADs for patients worldwide.

## 2. Improvements in LVAD Technology

### 2.1 Miniaturization of LVADs

The trend toward miniaturization in LVADs is driven by the need for less invasive implantation procedures, less physical burden on patients, improved patient quality of life, and the potential to address anatomical challenges in smaller patients [13]. Smaller devices reduce surgical trauma and the risk of complications, thus shortening recovery times. Advanced manufacturing techniques such as precision machining and three-dimensional (3D) printing enable the production of compact, intricate components without compromising functionality. These smaller LVADs also offer more placement options within the patient’s body, accommodate a wider range of anatomical variations, and reduce the physical burden on patients. LVAD miniaturization (Table 1) is exemplified by devices like the HeartMate 3, with its compact centrifugal-flow design [14], and the HeartWare Ventricular Assist Device (HVAD), recognized for its innovation during its time as one of the smallest full-support LVADs before being retired from the market in 2021. These designs facilitate both less invasive implantation and faster recovery times [15]. The Jarvik 2000 stands out for its small, lightweight build and adaptable anatomical configuration; these features provide flexibility in surgical approach during implantation, such as the choice of inflow and outflow cannulation sites [14]. The ReliantHeart aVAD [16] also exemplifies this trend toward miniaturization; precision machining and 3D printing are used to produce a device that is small but functional.

**Table 1.** **Notable improvements to LVAD technology**.

Improvement category	Specific improvement	Example devices/Components	Description
Miniaturization	Smaller device sizes	HeartMate 3	Compact centrifugal-flow design, less invasive implantation, quicker recovery
		HeartWare HVAD*	Small size facilitates less invasive implantation and faster recovery
		Jarvik 2000	Lightweight, adaptable configuration, advanced manufacturing techniques
		ReliantHeart aVAD	Precision machining, 3D printing, smaller size reduces physical burden
Passive coatings	DLC and MPC coatings	VentrAssist LVAD	DLC coatings reduce thrombosis but are prone to micro-cracks and delamination
		EvaHeart LVAD	MPC polymer coatings for hemocompatibility; biodegradability limits long-term antithrombogenic effectiveness
	Textured surfaces	HeartMate family	Promotes endothelial cell adhesion, forms stable neointimal lining, reduces thrombosis and infection risk
Active coatings	Heparinization	Carmeda BioActive surface	Covalently bonded heparin, longer-lasting anticoagulant activity, no heparin consumption
		Trillium	Similar to Carmeda, provides long-lasting anticoagulant activity
Design improvements	Optimized geometry	HeartAssist5	Ultrasonic flow probe, optimized pump geometry, highly polished titanium surfaces, Carmeda BioActive Surface coating
	Reduced shear stress	DuraHeart LVAD	Centrifugal-flow rotary pump, magnetically suspended impeller, large gaps, stable heparin coating
	Enhanced blood flow	HeartWare HVAD*	Magnetic suspension, hydrodynamic thrust bearings, integrated inflow cannula, titanium and thermoplastic polyester ether ketone materials
	Reduced thrombosis	HeartMate 3	Full magnetic levitation, wide blood flow passages, speed modulation, sintered titanium microspheres for endothelial tissue interface
Shape memory alloys	NiTi alloy properties	ParaCor HeartNet	Uses nitinol for flexible support, optimal stress-strain balance, ease of implantation and removal

LVAD, left ventricular assist device; 3D, three-dimensional; DLC, diamond-like carbon; MPC, 2-methacryloyloxyethyl phosphorylcholine; NiTi, nickel-titanium. *No longer commercially available.

### 2.2 Surface Coatings

Surface coatings have become a pivotal area of development in LVAD technology. These coatings are designed to enhance biocompatibility and reduce the risk of thrombosis and infection, which are significant concerns in long-term MCS [17]. Antithrombogenic coatings, such as heparin and hydrophilic polymers, create a hemocompatible [18] surface that minimizes blood clot formation. Additionally, antimicrobial coatings [19] incorporating silver ions or other antibacterial agents help prevent infections at the implant site. These advancements in surface technology not only improve the safety and effectiveness of the devices but also extend their operational lifespan by promoting biocompatibility, which reduces thrombotic complications, and by preventing infections, which minimizes the need for corrective interventions.

#### 2.2.1 Passive Coatings

Diamond-like carbon (DLC) coatings are widely used for the blood-contacting internal surfaces of devices like the VentrAssist LVAD [20] because of DLCs’ excellent mechanical, chemical, and electrical properties. DLC-coated surfaces interact minimally with the immune system, clotting factors, and platelets, making them effective in reducing thrombosis. However, they can develop micro-cracks and delamination due to internal stresses.

2-Methacryloyloxyethyl phosphorylcholine (MPC) polymer coatings enhance the biocompatibility of implantable devices by incorporating the phosphatidylcholine head group within its polymer backbone, which effectively suppresses thrombin formation and platelet activation [21]. The EvaHeart LVAD [22] uses MPC polymer coatings to maximize hemocompatibility. Although they are effective in the short term, MPC polymers are biodegradable, which limits their long-term antithrombogenic effectiveness.

Textured blood-contacting surfaces are more biocompatible than smooth ones because textured surfaces promote the adhesion of circulating endothelial cells to form a stable neointimal lining, thus reducing thromboembolic risk and the need for anticoagulation therapy. Techniques to create these surfaces include solid free-form machining, molding, and laser machining [23]. The various HeartMate LVADs have textured surfaces to minimize thrombosis and reduce infection risk.

#### 2.2.2 Active Coatings

Heparin is a popular surface coating for implantable devices due to its potent anticoagulant properties. Heparin binds to antithrombin-III, significantly enhancing its ability to inactivate factor Xa and thrombin, which are key mediators of thrombosis. Heparin can be attached to various substrates through ionic or covalent bonding. Ionic bonding can be used to attach heparin to the surfaces of implantable devices. However, there is a risk that heparin molecules may slowly release into the bloodstream over time [24]. Covalent bonding, used in products like Carmeda BioActive Surface and Trillium, provides longer-lasting anticoagulant activity without the heparin being consumed in the bonding reaction [25]. However, heparin has limitations, including its lack of antiplatelet activity and finite lifespan.

Researchers have experimented with seeding VAD surfaces with endothelial cells, which release biologically active agents that limit thrombogenesis and therefore could enhance a VAD’s biocompatibility [26]. However, the usefulness of this approach is controversial due to challenges with cell extraction, texturing surfaces, and maintaining cell function over time.

### 2.3 Other Design Improvements

The HeartAssist5 LVAD features an integrated ultrasonic flow probe that provides real-time blood flow measurements, and hemocompatibility is improved by the device’s optimized pump geometry, designed to reduce blood trauma and minimize the risk of thrombosis. The impeller, flow straightener, and diffuser design eliminate blood stagnation, thereby reducing thrombus formation [27, 28]. The device’s blood-contacting surfaces are highly polished titanium with a Carmeda BioActive Surface coating.

Another example of design improvement is the DuraHeart LVAD [29, 30]. This device uses a centrifugal-flow rotary pump with a magnetically suspended impeller for durability. The large gaps between the impeller and pump chamber walls improve washout and reduce shear stress. Blood-contacting surfaces are coated with stable, covalently bound heparin to enhance hemocompatibility.

The HVAD pump [31] features a magnetic suspension and hydrodynamic thrust bearings, which enhance blood flow and reduce blood trauma. Its integrated inflow cannula minimizes the pump footprint, allowing implantation within the pericardial space. The pump is made of titanium and thermoplastic polyester ether ketone, materials chosen for their superior durability and biocompatibility compared with the materials used in earlier pumps, such as stainless steel and polyurethane, which were more prone to wear and complications over time.

The HeartMate 3 uses full magnetic levitation and wide blood-flow passages to reduce shear stress. Preprogrammed speed modulation mimics a physiological pulse [32]. The device’s sintered titanium microspheres create an endothelial tissue interface, mitigating thrombosis risk.

Newer LVADs in development, such as the Toroidal Ventricular Assist Device (TORVAD), are currently undergoing animal trials and represent a significant evolution in device design. The TORVAD uses a unique toroidal design [33] with two independently controlled pistons that generate pulsatile flow, mimicking the natural heartbeat more closely than continuous-flow pumps. Operating at low rotational speeds [34], the TORVAD significantly reduces fluid shear stress, thus minimizing blood trauma. Its smart pumping technology allows the device to adjust flow in real time based on heart rate and blood pressure, optimizing patient outcomes and avoiding some of the complications associated with continuous-flow devices.

Each LVAD has unique characteristics because each of them is the product of a different wave of technological advancements. Table 2 provides a comparison of past and present LVADs, illustrating their advantages and limitations.

**Table 2.** **Advantages and disadvantages of present and past LVADs**.

LVAD	Type	Advantages	Disadvantages
Jarvik 2000	Axial flow	- Compact design	- Fully reliant on battery
		- Post-auricular driveline allows greater mobility	- Controller and batteries not waterproof
		- Adjustable speed settings	- Limited clinical adoption
HeartMate II	Axial flow	- Widely adopted and clinically proven	- Risk of pump thrombosis and gastrointestinal bleeding
		- Smaller than many centrifugal devices, fits under diaphragm	
HeartMate 3	Centrifugal flow	- Fully magnetically levitated rotor minimizes blood cell damage	- Requires sternotomy or thoracotomy for implantation
		- Low rates of thrombosis and bleeding	- Complex device management with external controllers
		- Suitable for bridge to transplant and destination therapy	
ReliantHeart aVAD	Axial flow	- Lightweight (92 g)	- Still investigational in the US
		- Fits within pericardium without needing a subdiaphragmatic pocket	- External power sources and controller might limit patient mobility
		- Allows pulse transfer from the native heart	
VentrAssist	Centrifugal flow	- Magnetically levitated impeller reduces mechanical wear	- Bulkier design than newer models
		- Designed to reduce thrombosis and hemolysis	- Only limited long-term data available
Evaheart	Centrifugal flow	- Excellent durability with low rates of thrombosis and bleeding	- Larger device, making it less suitable for small patients
		- Large pump output suitable for larger patients	- Surgical implantation is more complex
HeartAssist5	Axial flow	- Compact size	- Requires a large external controller
		- Features dual cannula options for better fit	- Less clinical experience than more established devices
DuraHeart	Centrifugal flow	- Hybrid magnetic and hydrodynamic levitation for minimal wear	- Bulky external components
			- Limited adoption (due to competition from other centrifugal pumps) limits available clinical data
HeartWare HVAD	Centrifugal flow	- Small and compact, suitable for sternotomy or thoracotomy	- External driveline increases infection risk
		- Lower rates of thrombosis than axial-flow devices	- Withdrawn from the market in 2021 due to safety concerns with pump thrombosis and neurological events

LVAD, left ventricular assist device.

## 3. Wireless VADs

A common complication of LVADs is driveline infection (DLI) [35]. When a driveline is used to connect the pump to an external power source, the driveline penetrates the skin, creating a pathway for bacteria to enter. Risk factors for DLI include older age, diabetes, renal failure, malnutrition, increased patient activity, and obesity [36]. DLI can lead to various other infectious complications, such as pump pocket infection, endocarditis, and mediastinitis. The REMATCH trial [37] highlighted infection as a major cause of mortality in LVAD patients, accounting for 41% of deaths.

Furthermore, DLI incurs substantial costs; a subanalysis [38] of REMATCH trial data identified sepsis, pump housing infection, and perioperative bleeding as principal drivers of cost for LVAD recipients during their hospitalization. The predicted cost of implantation varied substantially with the presence and severity of complications; estimates ranged from $119,874 for uncomplicated cases to $869,199 for cases involving multiple complications. Infections can incur further costs if they necessitate readmission; the average cost of readmission for LVAD patients ranges from $30,627 to $61,569 per readmission, with an average total cost of $105,326 per patient during the first year of MCS [38]. These costs encompass hospitalization, outpatient regimens, and associated medical interventions. The financial implications of VAD-related infection extend beyond direct medical expenses, including indirect costs such as reduced quality of life, lost productivity, and caregiver burden.

Current methods to reduce DLIs, such as decreasing driveline diameter [39] and coating surfaces with antibiotics [40], are only moderately effective. A better solution may be transitioning to VADs that use wireless power transfer instead of a driveline. Wireless power transfer would make these VADs fully implantable, thereby significantly reducing the potential for infection and improving overall safety and quality of life for patients requiring long-term cardiac support. Examples of current wirelessly powered devices are shown in Table 3 (Ref. [41]).

**Table 3.** **Ventricular assist devices with wireless power**.

Device	Technology type	Description
Corvion VAD	Integrated batteries	Incorporates a small internal battery and controller, allowing patients to be free from external power sources for up to 12 hours
Abbott FILVAS	Resonant coupling	Uses Resonant Link’s wireless power transfer technology for safe and efficient energy transfer; aiming for a fully implantable system
Medtronic fully implantable LVAD	TET systems	Limited information available, but working toward a fully implanted wireless LVAD using TET systems
Jarvik 2000 FlowMaker/Leviticus Cardio	Magnetic induction	Features the wireless Coplanar Energy Transfer system, which provides uninterrupted power to fully implanted LVADs; an external power unit is worn on the body or placed near the patient, enhancing mobility and comfort
HeartMate 3	Experimental wireless power modules	Wireless power modules inductively couple power from an external transmitter coil to an internal receiver coil, reducing the need for direct skin breaches
Hybrid pump developed by Horie *et al*. [41]	Hybrid pump systems	Combines magnetic and electric pumps in a single unit with a magnetic torque transmission system that charges wirelessly without an external coil, ensuring stable power transmission

VAD, ventricular assist device; FILVAS, Fully Implantable Left Ventricular Assist System; LVAD, left ventricular assist device; TET, transcutaneous energy transmission.

The concept of transcutaneous energy transmission (TET) systems encompasses technologies used in various experimental and commercial VADs to wirelessly transfer power through intact skin [42]. These systems typically use magnetic induction or resonant coupling to transmit power from an external source to an internal receiver coil, which then converts the energy to electrical power for the VAD. Efforts by multiple companies and research institutions are ongoing to perfect this technology, aiming to make VADs completely wireless and thereby significantly improve quality of life and safety for patients requiring long-term cardiac support.

Corvion’s VAD (formerly known as the Everheart System) [43] incorporates a small internal battery and controller, allowing patients to be free from external devices for up to 12 hours. Abbott’s Fully Implantable Left Ventricular Assist System (FILVAS) uses Resonant Link’s wireless power transfer technology, ensuring safe and efficient energy transfer [44]. Medtronic is also working on a fully implantable LVAD [45], although only limited information is available about their progress.

Among older notable examples is the Jarvik 2000 FlowMaker [12]. This VAD uses Coplanar Energy Transfer (CET), a TET system developed by Leviticus Cardio [46]. The CET system is designed to provide uninterrupted power wirelessly to fully implanted LVADs [47]. It includes an external power unit that can be worn on the body or placed near the patient, transmitting energy through the skin to an implanted receiver that powers the LVAD. This technology reduces infection risk and improves patient mobility and comfort. Likewise, the Jarvik 2000 is a compact axial-flow LVAD whose percutaneous driveline has been replaced with a TET system.

Another innovative example is the HeartMate 3, which primarily operates with a driveline but whose manufacturer has experimented with a wireless power module. The module operates by inductively coupling power from an external transmitter coil to an internal receiver coil [48].

A hybrid approach using two separate pumps [49]—one magnetic and one electric—has been studied, but the initial version proved to be space-consuming, and the distance had to be short between the extracorporeal magnets and the intrathoracic pump. To overcome these problems, Horie *et al*. [41] developed an innovative single hybrid pump that can operate by either magnetic or electric force and that can be charged wirelessly without an external coil. The hybrid pump features a magnetic torque transmission system to address distance limitations and ensure stable power transmission. This centrifugal pump includes neodymium magnets that drive the pump in both modes and also function as a generator to charge an internal battery while the pump is in magnetic mode. The mode-switching system utilizes the impeller’s rotational speed to determine the optimal mode. For instance, the system might employ the magnetic mode at lower speeds for efficient, low-power operation and switch to the electric mode at higher speeds for increased outflow. However, identifying the ideal speed thresholds for mode switching requires further investigation.

## 4. Minimally Invasive Surgical Implantation Techniques

Historically, the most common approach for VAD implantation was median sternotomy, a tried-and-true procedure that, compared with other methods, offered excellent exposure for pump insertion, straightforward access to the left ventricle, and provided an excellent anatomical overview. However, this approach posed a risk of postoperative bleeding, infection, and sternal instability. This was especially true for early VADs, whose large size necessitated an extended sternotomy.

The transition from pulsatile to continuous-flow pumps revolutionized the development of MCS because it allowed VAD designs to be more compact. Not only has the development of these smaller LVADs reduced the invasiveness of the standard implantation procedure (because implanting a smaller pump allows a smaller incision), but it has also driven the development of less invasive surgical techniques for implanting these devices [50, 51].

The miniaturization of LVADs has significantly advanced the field of MCS; notable examples include the HeartWare HVAD, HeartMate II, HeartMate 3, and Jarvik 2000 [52]. These devices have evolved to address the challenges of implantation and patient care. The HeartWare HVAD, a centrifugal pump with a more compact design than its predecessors, can be implanted via a thoracotomy rather than a full median sternotomy [31]. Similarly, the HeartMate II and HeartMate 3, with their progressively refined designs, are also implantable by thoracotomy. The HeartMate 3, with its innovative features, remains on the market, continuing to support advanced HF patients. The HeartMate II is still available, as well. In contrast, the Jarvik 2000, an early-generation device known for its compact design, has been phased out, and the HVAD was discontinued in 2021. The Jarvik 2000, while innovative for its time, lacks the technological advancements seen in newer devices, such as fully magnetically levitated rotors, which reduce blood cell damage. The trend toward miniaturization reflects a broader effort to enhance device functionality and implantability while improving patient outcomes [53].

The choice of surgical approach is crucial for optimizing patient outcomes and minimizing complications [54]. The left thoracotomy is commonly used for pump inflow insertion, providing access to the left ventricle and enabling secure placement of the inflow cannula. This approach allows surgeons to position the cannula to ensure optimal blood flow from the heart to the LVAD. For the outflow graft placement, a right thoracotomy or upper mini-sternotomy is typically used, depending on the specific device and surgeon preference. The right thoracotomy offers access to the ascending aorta for outflow graft connection, allowing the blood to be efficiently redirected into the arterial system. Alternatively, the upper mini-sternotomy provides a less invasive access point while still allowing precise placement of the outflow graft in the ascending aorta [55]. Both techniques aim to balance reduced surgical invasiveness, and the choice between them often depends on the surgeon’s experience, the patient’s anatomy, and the specific LVAD being implanted.

Minimally invasive approaches, such as hemisternotomy combined with left anterolateral thoracotomy, or right mini-thoracotomy with left anterolateral thoracotomy, aim to minimize sternal trauma and preserve pericardial integrity [56]. These techniques have been associated with several benefits: reduced intraoperative bleeding, fewer blood transfusions, shorter intensive care unit and hospital stays, and lower rates of right ventricular failure (RVF) [57]. The preservation of the pericardium helps maintain anatomical support for the heart, potentially reducing the incidence of RVF—a common complication in LVAD implantation.

Further, minimally invasive techniques have shown advantages in reoperations, including pump exchanges, wherein the reduced surgical trauma minimizes complications and supports quicker recovery [58]. The multicenter LATERAL trial confirms that less invasive LVAD implantation yields comparable or even better outcomes than traditional sternotomy approaches, including lower rates of adverse events and postoperative complications [59].

To avoid complete sternotomy, new implantation techniques have been devised whose development has been facilitated by advancements in off-pump LVAD implantation techniques. When VADs are implanted, patients are usually on cardiopulmonary bypass (CPB). However, ample evidence links VAD implantation under CPB to several detrimental outcomes, such as coagulopathy, elevated pulmonary vascular resistance, platelet activation, and compromised renal function. Implanting VADs without the use of CPB may help to reduce these postoperative problems without compromising hemodynamics or causing excessive bleeding during implantation. A study of 25 patients with LVADs implanted off-pump showed that using an off-pump technique has short-term benefits such as minimizing perioperative bleeding, the need for reoperation to address blood loss, acute renal injury, and respiratory problems [60]. On the other hand, off-pump VAD implantation is typically only advised in specific circumstances because it still incurs the risk of air embolism, stroke, blood loss, and right HF [51, 60, 61].

Another approach to avoiding complete sternotomy is developing less invasive access methods for LVAD implantation. The traditional LVAD insertion procedure has three phases: insertion of the inflow cannula and pump, anastomosis of the outflow graft, and driveline tunneling; only the first two phases can be modified for use in minimally invasive procedures. A left thoracotomy, which involves making an incision on the left side of the chest, is the best and most common approach in these less invasive techniques. This approach provides effective access to the heart and surrounding structures while causing less trauma than a full sternotomy. This method is most often used in patients being bridged to heart transplant because it avoids the sternal incision that will be needed for the transplant.

Patients’ long-term results are significantly influenced by the outflow graft’s location. Incorrect placement creates blood flow turbulence, which increases the risk of outflow graft thrombosis. Fortunately, several options are available for placing the outflow graft. The surgical standard consists of an anastomosis to the ascending aorta through a thoracotomy approach; less common outflow sites include the descending aorta, the axillary and subclavian arteries, and the supraceliac abdominal aorta [62, 63].

Several studies have been performed to determine whether it is advantageous to use minimally invasive techniques for VAD implantation. The LATERAL trial [59] underscores the significance of the thoracotomy approach for LVAD implantation, offering compelling evidence for its benefits over traditional sternotomy. The results highlight that LVAD implantation performed through a lateral thoracotomy or upper hemisternotomy provides excellent visualization of cardiac structures while minimizing surgical trauma. It is noninferior in terms of 6-month survival and significantly shortens hospital stays compared with sternotomy. Importantly, the long-term data from the trial reveal low rates of adverse events such as driveline infection, gastrointestinal bleeding, and stroke, the risk of which decreases over time after LVAD implantation. The thoracotomy approach not only enhances patient recovery and quality of life but also supports prolonged LVAD therapy amidst a growing shortage of donor hearts and extended wait times for transplantation. One study examined the results of 60 LVAD implantations: 30 by mini-thoracotomy and 30 via complete sternotomy. Mini-thoracotomy was associated with a lower death rate, shorter CPB times, less drainage, and less blood product infusion. Hospital length of stay did not differ significantly between groups; however, the mini-thoracotomy group had much shorter intensive care unit stays and extubation times. In a similar study of LVAD recipients performed in 2018, 10 patients who underwent a full sternotomy were compared with 32 patients who underwent various less invasive surgical techniques: left mini-thoracotomy with outflow graft anastomosis to the left axillary artery (n = 5), right and left mini-thoracotomy (n = 7), and left mini-thoracotomy with upper mini-sternotomy (n = 20). Findings revealed that after the less invasive surgical procedures, the survival of severely impaired patients (who comprised 77% of the patients in the less invasive group) was largely satisfactory. The left anterior mini-thoracotomy with outflow anastomosis to the left axillary artery had the best outcomes in the overall study. These results indicate that minimally invasive procedures could be used to implant LVADs just as effectively as the traditional full sternotomy method while reducing various risks involved [64, 65, 66].

Various studies suggest that for LVAD implantation, less invasive surgical techniques are a safe, but not always better, option than median sternotomy. In a study performed in 2022, 162 patients underwent median sternotomy, and 60 patients underwent lateral thoracotomy for LVAD implantation. Both perioperative use of blood products and early right ventricular failure were significantly less frequent in patients who had lateral thoracotomy, indicating that the less invasive surgical technique can be safely used even in the sickest patients. Even with these benefits, less invasive methods are challenging to use because smaller incisions cannot be used for concurrent operations (e.g., coronary artery bypass grafting, mitral valve replacement), potentially leaving unresolved other cardiac issues that could increase the patient’s risk of requiring readmission to treat volume overload and subsequent tricuspid regurgitation [67].

As previously mentioned, the development of third-generation LVADs, which feature continuous-flow technology and are smaller, more durable, and more efficient than earlier versions, has resulted in notable advancements in surgical techniques. New technological advancements will be necessary for any groundbreaking new techniques. Nevertheless, an experimental robotic-assisted VAD implantation technique has been shown to be feasible; it involves a small thoracotomy and the DaVinci robotic system, which can be used to execute anastomoses. Although this procedure is novel, the costs associated with it probably outweigh the benefits. However, more sophisticated robotic-guided implantation may prove to be a viable strategy in the future. Additionally, further miniaturization of devices, better insertion tools, and completely implantable VADs are some of the technological advancements that are anticipated to revolutionize minimally invasive surgical practices [68, 69, 70].

### Valvulopathy and LVADs

The interplay between valvulopathy and LVADs is intricate, reflecting the complexity of managing advanced HF with MCS [71, 72]. During LVAD implantation, continuous-flow devices create a transvalvular pressure gradient across the aortic valve, which can alter aortic valve function. Specifically, this persistent pressure gradient often results in the aortic valve remaining closed throughout the cardiac cycle, potentially exacerbating pre-existing aortic insufficiency (AI) or causing de novo AI to develop [73]. In patients receiving LVAD support, AI creates a closed-loop circulation between the ascending aorta and left ventricle, which can compromise left ventricular (LV) unloading, reduce peripheral perfusion, and worsen HF symptoms. A meta-analysis has shown that significant AI can develop in up to 25% of patients, increasing their risk of rehospitalization and mortality [74].

To manage AI, concomitant surgical interventions at the time of LVAD implantation include aortic valve repair, replacement, and closure, which have varying degrees of effectiveness and different associated risks. AI may develop or worsen after LVAD placement, and although conventional surgical treatments are available, percutaneous transcatheter interventions like transcatheter aortic valve replacement and placing occlusion devices have shown promise in reducing severe AI with less procedural risk. Preventive measures, including optimizing pump speed and maintaining strict blood pressure control, are crucial in managing AI and improving outcomes [71].

Mitral regurgitation (MR) results from ventricular remodeling and is associated with poorer outcomes after LVAD placement. Conventional treatments, including pharmacological management and cardiac resynchronization therapy, can reduce MR severity [75]. Percutaneous mitral valve repair has shown mixed results but may benefit some patients with severe MR and LV dysfunction. Surgical mitral valve repair or replacement is an option but is not universally recommended because it has no proven survival benefit in routine cases [76].

LVAD implantation often alleviates MR, making concurrent mitral valve surgery unnecessary in many cases [77]. Data suggest that residual MR after LVAD implantation does not consistently affect long-term survival, although it correlates with worse right ventricular function and greater risk of rehospitalization as shown in a meta-analysis [78]. Mitral stenosis and prosthetic mitral valves generally do not contraindicate LVAD implantation, but mitral stenosis should be addressed if severe, as it impairs LV filling.

Tricuspid regurgitation (TR) is prevalent in HF patients. It worsens with HF severity and is associated with greater morbidity and mortality risk. Functional TR often results from right ventricular remodeling and dilation due to left-sided heart dysfunction or pulmonary hypertension [79]. In LVAD-supported patients, TR typically improves within the first month as pulmonary pressures decrease, but significant TR complicates the early postoperative course and can necessitate additional support or prolonged intensive care unit stays [80].

Although tricuspid valve repair is frequently performed at the time of LVAD implantation, the evidence supporting its benefit is mixed [81]. Some studies suggest that repairing TR may reduce patients’ risk of early right HF and rehospitalization, but the overall survival benefits are unclear. Larger registry analyses and recent trials have not demonstrated clear advantages of concurrent tricuspid valve procedures, with some associating these operations with greater risks and complication rates [81]. Current guidelines recommend careful reevaluation of TR after LVAD placement and individualized decision-making for tricuspid valve intervention; ongoing research is needed to clarify which patients might benefit from concomitant surgery [82].

## 5. Pediatric VADs

The development of VADs for children is still behind that of adult VADs, even though it is a seriously unmet need. However, given the substantial advancements made in pump design, greater understanding of how LVAD implantation should be timed, and improved post-implantation management, particularly regarding blood pressure control and anticoagulation, the gap between pediatric and adult VAD technology is anticipated to narrow soon. In the current field of pediatric VAD therapy, numerous goals still must be met, such as devising ways to identify patients who would benefit the most from VAD support and to choose the right device for the patient according to their unique clinical situation and device complication profile [83].

Plenty of evidence supports the need for better pediatric VAD technology. In a 2006 study conducted on behalf of the Pediatric Heart Transplant Society, researchers assessed children (median age, 13.3 years) who were bridged to transplantation with a VAD. The results showed that 86% of children who received VAD support were successfully bridged to transplantation; however, those with congenital cardiac disease and smaller size had worse outcomes, often dying while awaiting a transplant [84]. In another multicenter trial, 204 children received the Berlin EXCOR VAD, a device specifically designed for pediatric patients, at 47 locations between 2007 and 2010. Notably, while receiving Berlin EXCOR support (median duration, 70 days), more than two thirds of patients who weighed less than 5 kg died.

Patient-device size mismatch and the underlying causes of a pediatric patient’s HF are two possible reasons for such poor outcomes. Because an LVAD’s flow rate is associated with both the right ventricle’s cardiac output and the size of the pump, an LVAD sized for a small child may produce too little flow, causing blood to stagnate and resulting in pump thrombosis [85, 86]. Also, VAD support may be problematic in pediatric patients whose HF is due to congenital heart disease, particularly if they have single-ventricle physiology [87]. A recent study found that survival rates were much lower for patients with single-ventricle physiology (42%) than for patients with biventricular physiology (73%) [88]. These results demonstrate the challenges of supporting this patient population with VADs.

The trend of selecting continuous-flow VADs in children was further accelerated by the introduction of a smaller continuous-flow device: the HVAD. However, eligibility for HVAD support in children was previously established for those with a body surface area as small as 0.6 m^2^. The discontinuation of the HVAD has left a gap in available pediatric treatment options, highlighting the urgent need for alternative solutions to address the persistent challenge of patient-device size mismatch. Efforts to improve outcomes in pediatric patients with end-stage HF must now focus on developing and refining new devices specifically designed for smaller bodies. Many implantable continuous-flow VADs suitable for small children are currently undergoing testing, such as a pediatric version of the HeartMate 3, the HeartWare Miniature Ventricular Assist Device (MVAD), and the Jarvik Infant [86, 89]. These upcoming devices could provide much-needed MCS options for pediatric patients with end-stage HF.

## 6. RVAD Support

Although LVADs can significantly improve cardiac output by increasing blood flow, this improved flow can increase venous return to the right ventricle, placing a higher volume load on it—sometimes to the point of right ventricular overload [53].

Improved LV flow can negatively affect the right ventricle in other ways. The interventricular septum may shift toward the left ventricle, distorting right ventricular geometry and impairing the ventricle’s filling and contraction [90]. These changes can result in clinical signs of right ventricular failure, such as peripheral edema, hepatomegaly, ascites, and jugular venous distention. Understanding these mechanisms is crucial for managing patients with LVADs and addressing the potential need for RVAD support in such cases.

Right ventricular failure frequently occurs secondary to LV dysfunction, resulting in a greater need for combined support than for isolated RVAD use. Only rarely does a patient require implantation of an RVAD without also requiring, or already being on, LVAD support. This is because LV failure is more common and typically more severe than right ventricular failure [91].

Acute right ventricular failure can arise from various conditions such as right ventricular myocardial infarction, pulmonary embolism, and acute decompensation in patients with chronic pulmonary hypertension [92]. In these instances, the right ventricle can fail abruptly, necessitating temporary RVAD support to stabilize the patient and allow the right ventricle time to recover. Additionally, after cardiac operations—particularly those involving the right side of the heart—and procedures such as heart or lung transplantation, patients might have right ventricular failure. In these postoperative situations [93], an RVAD can be used to provide necessary support until the right ventricle regains adequate function.

Another scenario for isolated RVAD implantation involves patients with congenital heart defects [94]. These patients may have anatomical or functional abnormalities predominantly affecting the right side of the heart, leading to right ventricular failure while the left ventricle remains largely functional.

The development of RVADs [95] began in the mid-20th century, paralleling advances in LVAD technology. Early RVADs were adapted from LVAD designs, often resulting in bulky and cumbersome devices that were not ideal for the lower-pressure, higher-volume demands of the right ventricle. Initial RVADs were extracorporeal pumps connected to the patient via large cannulas. These systems, such as the Abiomed BVS 5000, provided necessary support [96] but were limited by their large size, risk of infection, and patient immobility. Despite their limitations, these early RVADs paved the way for more sophisticated designs. One example is the CentriMag System, which consists of an extracorporeal centrifugal pump connected to the right atrium and pulmonary artery [97]. This system provides continuous MCS to the right ventricle, helping to alleviate right HF in patients with an acute condition such as postcardiotomy shock or acute myocardial infarction. The CentriMag’s extracorporeal design allows rapid implantation and adjustment, making it suitable for emergency situations that necessitate immediate support.

The TandemHeart RVAD is another extracorporeal device designed to provide temporary support to the right ventricle [98]. It consists of a centrifugal pump connected to the femoral vein and artery, with inflow from the right atrium and outflow to the pulmonary artery. The TandemHeart RVAD is often used in conjunction with other temporary MCS devices to provide comprehensive circulatory support in critically ill patients. Its flexible design allows extensive customization for easy adjustment and adaptation to individual patient needs. The evolution of this device led to the development of the LifeSPARC system, which retained the TandemHeart’s key features while adding improvements in pump durability, ease of use, and patient outcomes. LifeSPARC can be used in both right and left ventricular support, making it versatile for various clinical scenarios [99].

The shift toward implantable devices was a significant milestone in RVAD innovation. This shift was accomplished by focusing on the development of smaller, more efficient pumps and cannulas tailored to the requirements of the right side of the heart. The ProtekDuo cannula is a notable advancement in this area, having a dual-chamber design that can provide simultaneous support to both the right atrium and the right ventricle or temporarily support the left ventricle [100] if necessary. The device’s small size and full implantability enhance patient mobility and comfort [101].

Another percutaneous device is the Impella RP, which is a critical option for patients in right ventricular (RV) failure and especially those with RV-predominant cardiogenic shock. This device offers a percutaneous means of providing circulatory support by bypassing the right ventricle, thereby helping to stabilize patients who are unresponsive to medical therapy. The Impella RP facilitates rapid hemodynamic improvement, protecting the myocardium from ischemia and promoting recovery of RV function [102].

The HeartMate 3 and the HeartWare HVAD LVADs represent two innovative options for use as RVADs, offering long-term MCS for patients with advanced HF. Both devices are fully implantable within the chest cavity, eliminating the need for an external driveline and enhancing patient comfort and mobility. Their compact size allows relatively straightforward surgical implantation, although the choice of specific implantation technique depends on patient anatomy and surgeon preference [103].

For use as an RVAD, the HeartMate 3 needs to be modified. A group shared their technique of adding a polytetrafluoroethylene ring to the inflow cannula to reduce the intraluminal part of the cannula [104]. In a case reported by Ricklefs *et al*. [105], a 72-year-old man with isolated right HF secondary to arrhythmogenic right ventricular cardiomyopathy underwent implantation of a HeartMate 3 as an RVAD. The surgical team adapted the device for right heart support—a procedure that presented several challenges due to the anatomical and functional differences between the left and right ventricles. To mitigate the risk of suction events from the device’s inflow cannula, several layers of felt pledgets were sutured onto the sewing ring before it was positioned on the right atrium. Additionally, the outflow graft was modified by reducing its diameter from 14 mm to 10 mm. The resulting greater resistance allowed the pump to operate at a higher rotational speed, thus reducing the risk of thrombus formation. Postoperative echocardiography showed a marked reduction in RV dilation and stable LV function.

Another group, which used the HVAD as an RVAD, showed that right atrial cannulation with tricuspid valvectomy makes the HVAD technically easier to implant than the HeartMate 3 [106].

The retirement [107] of the HeartWare HVAD from the MCS market is a substantial loss due to its reputation for reliable performance, miniaturized pump design, and suitability for patients with smaller chest cavities. As one of the leading VADs, the HVAD provided an option for long-term MCS that improved patient outcomes and quality of life. Its discontinuation not only reduces the diversity of available options for patients and clinicians but also represents a setback in ongoing research and development efforts in the field of MCS, as it revealed new engineering obstacles that future VAD designs must overcome. Clinicians must now adapt to the absence of a once widely used device by changing their clinical protocols and patient management strategies.

## 7. Cost-effective VADs (VAD Access in Lower-Income Countries)

The prohibitive costs [108] associated with traditional LVADs have created substantial disparities in access to life-saving therapies, particularly in low- and middle-income countries. Acknowledging this pressing issue, researchers and innovators outside the United States have spearheaded the development of cost-effective MCS devices to bridge the gap in patient care.

One such device is the paracorporeal pulsatile ventricular assist device developed by Vitalmex in Mexico City. This device represents a groundbreaking advancement in cost-effective MCS devices [109]. This device combines pulsatile-flow technology with a paracorporeal design, offering effective circulatory support while minimizing manufacturing costs. Their pump’s housing is innovative because it allows structural and pneumatic connection resistance, which makes handling during implantation more intuitive. Thus, implanting the device requires no specialized training for surgeons in developing countries, who may have no experience with LVAD surgery. It is designed with a focus on material efficiency and functional configuration, making it both lightweight and highly effective. Central to this system is a 500-gram reusable pump housing paired with a disposable pump insert, inflow and outflow cannulas, and a pneumatic driver [110].

The disposable insert features a transparent, oblate-spherical bladder made from silicone-elastic material with a volume capacity of 65 cm^3^. This bladder is engineered for durability and flexibility, ensuring reliable performance during each cardiac cycle. It is securely connected to two titanium tubes, which provide structural support and integrate seamlessly with the trileaflet silicone-elastic inlet and outlet valves. These valves are critical for maintaining unidirectional blood flow and preventing backflow.

The insert is designed to lock precisely into place between the two halves of a rigid, reusable polycarbonate shell, which forms an airtight seal. This polycarbonate shell is not only durable but also lightweight, contributing to the overall portability and efficiency of the system.

The system uses a pneumatic driver that delivers compressed air through a six-foot segment of flexible tubing. This air is delivered in bursts, pressurizing the airtight pump housing and compressing the silicone-elastic bladder. As the bladder compresses, it ejects blood through the unidirectional outflow valve. When the pressure is released, the bladder re-expands, allowing blood from the left ventricle to fill it through the one-way inflow valve.

This configuration, with its emphasis on advanced materials like silicone-elastic and titanium, combined with the efficient design of the polycarbonate shell and pneumatic driver, underscores the Vitalmex system’s innovative approach to cardiac support.

Another group of Romanian researchers combined mathematical modeling, turbomachinery design, computational fluid dynamics (CFD), and 3D printing techniques to create a cost-effective solution [111] for advanced HF patients. The core of the device is its rim-driven, hubless axial-flow pump. This design choice is central to its cost-efficiency and performance. The pump components are crafted from resins and polycarbonate filaments by stereolithography 3D printing. These materials are less expensive than those used in conventional LVADs but still provide sufficient durability and biocompatibility.

Mathematical modeling played a crucial role in determining the optimal pump dimensions and operational parameters. The team calculated specific work, impeller diameters, and rotational speeds to achieve efficient blood flow and pressure. This modeling guided the preliminary design, which was then refined through CFD simulations. These simulations confirmed that the 11-mm–diameter pump could achieve a flow rate of 10 L/min at a rotational speed of 16,758 rpm, with minimal turbulence and pressure drop.

The design incorporates a wireless power supply system that uses electromagnetic induction to charge an internal battery, which eliminates the risk of infection associated with the percutaneous drivelines of traditional LVADs. The device is implanted subcutaneously, thus enhancing patient comfort and reducing infection risk.

## 8. Conclusions

The evolution of VADs constitutes significant advancement in managing HF, offering hope to both adult and pediatric HF populations. Continuous-flow VADs have significantly improved patient outcomes, yet they come with complications like DLIs and RV overload, necessitating further innovations.

Transitioning to wireless VADs holds promise for enhancing patient quality of life by eliminating the need for percutaneous drivelines and thus eliminating DLIs. Devices such as the Corvion VAD, Abbott FILVAS, and Jarvik 2000 FlowMaker illustrate ongoing advancements toward fully implantable systems. Additionally, minimally invasive surgical techniques for VAD implantation reduce postoperative complications and recovery time, making these devices more accessible and safer for patients.

The development of pediatric VADs lags behind the development of their adult counterparts but is critical for treating children with HF. Despite challenges such as patient-device size mismatch and congenital heart disease, continuous efforts in developing pediatric-specific devices, like the HeartMate 3 and Jarvik Infant, are paving the way for better outcomes.

RVADs are essential for managing right ventricular failure, often secondary to LV dysfunction. Advanced devices like the HeartMate 3 and HeartWare HVAD have been adapted for RV support, highlighting the need for tailored solutions in complex cardiac conditions.

The high cost of traditional LVADs has limited access to life-saving therapies in low- and middle-income countries. Innovations such as the paracorporeal device by Vitalmex and a 3D-printed hubless pump from Romania are designed to reduce costs and expand accessibility, aiming to bridge gaps in heart failure treatment globally. The ongoing advancements in VAD technology, from wireless power transfer and minimally invasive techniques to pediatric-specific designs and cost-effective solutions, reflect a dynamic field dedicated to improving patient outcomes and accessibility. Continued innovation and international collaboration are vital to addressing the diverse needs of HF patients worldwide by ensuring that VAD therapy becomes more effective, safer, and more widely available.
